# Isolation and Characterization of a Novel Strain of Mesenchymal Stem Cells from Mouse Umbilical Cord: Potential Application in Cell-Based Therapy

**DOI:** 10.1371/journal.pone.0074478

**Published:** 2013-08-26

**Authors:** Wen-Wen Li, Yau-Huei Wei, Hung Li, Dar-Ming Lai, Teng-Nan Lin

**Affiliations:** 1 Institute of Biochemistry and Molecular Biology, National Yang-Ming University, Taipei, Taiwan; 2 Department of Medicine, Mackay Medical College, New Taipei City, Taiwan; 3 Institute of Molecular Biology, Academia Sinica, Taipei, Taiwan; 4 Division of Neurosurgery, Department of Surgery, National Taiwan University Hospital, Taipei, Taiwan; 5 Institute of Neuroscience, National Yang-Ming University, Taipei, Taiwan; 6 Institute of Biomedical Sciences, Academia Sinica, Taipei, Taiwan; Georgia Regents University, United States of America

## Abstract

Human umbilical cord-derived mesenchymal stem cells (hUC-MSCs) have recently been recognized as a potential source for cell-based therapy in various preclinical animal models, such as Parkinson’s disease, cerebral ischemia, spinal cord injury, and liver failure; however, the precise cellular and molecular mechanisms underlying the beneficial outcomes remain under investigation. There is a growing concern regarding rejection and alteration of genetic code using this xenotransplantation approach. In this study, a novel strain of murine MSCs derived from the umbilical cord of wild-type and green fluorescent protein (GFP) transgenic mice have been successfully isolated, expanded, and characterized. After 10 passages, the mUC-MSCs developed a rather homogeneous, triangular, spindle-shaped morphology, and were sub-cultured up to 7 months (over 50 passages) without overt changes in morphology and doubling time. Cell surface markers are quite similar to MSCs isolated from other tissue origins as well as hUC-MSCs. These mUC-MSCs can differentiate into osteoblasts, adipocytes, neurons, and astrocytes *in vitro*, as well as hematopoietic lineage cells *in vivo*. mUC-MSCs also possess therapeutic potential against two disease models, focal ischemic stroke induced by middle cerebral artery occlusion (MCAo) and acute hepatic failure. Subtle differences in the expression of cytokine-related genes exist between mUC-MSCs and hUC-MSCs, which may retard and jeopardize the advance of cell therapy. Allografts of these newly established mUC-MSCs into various mouse disease models may deepen our insights into the development of more effective cell therapy regimens.

## Introduction

During the past decades, mesenchymal stem cells (MSCs) were the primary cell source for treating human-inherited and degenerative diseases with stem cell therapy owing to their wide availability, low tumorigenicity, and multipotent differentiation capability to mesodermal and non-mesodermal lineages, such as hepatocytes, neural cells, and pancreatic-like cells [1–4]. Furthermore, their low immunogenic nature and immunomodulatory capabilities make MSCs suitable for cell transplantation and treatment of immune disorders [5–9].

To date, MSCs have been successfully cultivated from various tissues, including bone marrow [10], circulating blood [11], trabecular bone [12], synovium [13], adipose tissue [14], placenta [15,16], heart [17], skeletal muscle [18], dental pulp [19], pancreas [20], and umbilical cord blood [21,22]. In 2004, Wang et al. demonstrated that human umbilical cord (hUC) itself also contains MSCs [23]. In contrast to other adult-type MSCs, several fascinating properties of fetal-type hUC-MSCs make them a more attractive candidate for clinical applications, including non-invasive harvest, higher cell number, and lower risk of viral contamination, aside from their basic feature of differentiating into tissues distinct from their origin *in vitro* and *in vivo* [24–26]. The hUC-MSCs appear to have higher proliferation potential, greater expansion capacity, and shorter doubling time compared to MSCs derived from other sources, likely due to their longer telomeres, greater telomerase activity, and higher telomerase reverse transcriptase activity [27–30]. Furthermore, the efficacy of induced pluripotent stem cells (iPSCs) derived from hUC-MSCs is much higher than other adult-type MSCs and fibroblasts [31].

Although an increasing number of reports have demonstrated the therapeutic effects of hUC-MSCs after transplantation in various experimental models, including Parkinson’s disease [32], cerebral ischemia [26,33,34], spinal cord injury [35], liver fibrosis [36], and bleomycin-induced lung injury [37], the precise cellular and molecular mechanisms underlying these beneficial effects of MSCs remain elusive and controversial. Despite similar characteristics in MSCs isolated from distinct tissue sources, recent studies have suggested that certain genetic or cellular variations do exist among tissue sources [38,39]. Moreover, several reports have pointed out that some differences exist between allogeneic and xenogeneic treatments in several animal models [40,41]. Therefore, it is conceivable that a xenogeneic treatment, such as hUC-MSCs transplanted into a rodent, may alter the host immune response, thereby leading to complications.

While UC-MSCs have been successfully isolated from the human, pig [42], cow [43], horse [44], canine [45], even chicken [46], MSCs from mouse umbilical cord have not been established. Mouse primary cell culture is an important platform for elucidating the molecular mechanisms of diseases; mice with various genetic manipulations have been used extensively in basic and preclinical research. Therefore, the establishment of mUC-MSCs for allogeneic cell therapy may deepen our insights into the underlying cellular and molecular mechanisms of various diseases. In this study, the mUC-MSCs derived from wild-type and GFP transgenic mice were successfully isolated, expanded, characterized, and differentiated into tissues distinct from their origin *in vitro*. The differentiation potential of hematopoietic lineage cells *in vivo* has also been evaluated by hematopoietic reconstitution. Moreover, two disease models, focal ischemic stroke induced by MCAo and acute hepatic failure, were employed to assess the therapeutic efficacy of mUC-MSCs.

## Materials and Methods

### Mice and Human UC-MSCs

The C57BL/6JNarl wild-type and C57BL/6-Tg(ACTB-EGFP) 1Osb/J transgenic mice used in this study were purchased from National Laboratory Animal Center (NLAC, Taipei, Taiwan) and the Jackson Laboratory (Bar Harbor, ME, USA), respectively. Studies were carried out in strict accordance with the recommendations in the guidelines for the Academia Sinica Institutional Animal Care and Utilization Committee (AS IACUC, http://iacuc.sinica.edu.tw/). All surgery was performed under isoflurane and chloral hydrate anesthesia, and all efforts were made to minimize suffering. Experimental procedures involving animals were approved by the AS IACUC (Permit Number: MMiRaIMBLH2007075, RMiIMBLH2009071, RMiRaIBMLT 2011066). The hUC-MSCs were kindly provided gratis from Dr. Woei-Cherng Shyu [26]. The culture condition and differentiation analysis of hUC-MSCs were followed as described previously [26].

### Isolation and Expansion of mUC-MSCs in vitro

Mouse umbilical cords were aseptically collected from C57BL/6JNarl and C57BL/6-Tg(ACTB-EGFP) 1Osb/J (GFP) mice. The collected umbilical cords were rinsed 3 times with phosphate-buffered saline (PBS, pH 7.4), once with DMEM-LG (Sigma-Aldrich, St. Louis, MO, USA), and transferred to one well of fibronectin (Millipore, Temecula, CA, USA) coated 24-well plate containing the culture medium. Next, each umbilical cord was mechanically cut with a pair of scissors into small pieces (0.5-1.0 mm), and incubated at 37 °C, 5% CO_2_ for 3-5 days to allow the cells to migrate from the explants. Then, the debris and floating cells were aspirated; the attached (plastic-adherent) cells were carefully rinsed with PBS, trypsinized by trypsin/EDTA, and transferred into one well of a fibronectin-coated 6-well plate. Serial passages were performed when cells reached confluence at a ratio of 1:1 from early passages (numbers 1-10). The heterogeneous cell populations derived from mouse umbilical cord appeared in early passages. The cellular morphology became homogenously spindle-shaped in cultures after 10 passages. Next, cells were seeded at a density of 1 × 10^5^ cells onto fibronectin-coated 100-mm tissue culture dishes at each passage. Single cell colonies originating from mUC-MSCs were also established using single cell per well dilution in a 96-well plate and microscopically monitoring of growth of each single cell-derived colony was performed daily. The cell from a single colony could be further serially transferred, expanded, and grown to confluence on 12-well, 6-well, and 100-mm culture dishes. The culture medium used for the growth of mUC-MSCs was modified from that used in a previous study [47]. The medium consisted of low serum and 56% DMEM-LG, 37% MCDB-201 (Sigma-Aldrich, St. Louis, MO, USA), with 1× insulin-transferrin-selenium (ITS; Gibco, Life Technologies, Carlsbad, CA, USA), 1× linoleic acid-bovine serum albumin (LA-BSA; Sigma-Aldrich, St. Louis, MO, USA), 0.1 mM ascorbic acid 2-phosphate (Sigma-Aldrich, St. Louis, MO, USA), 1 nM dexamethasone (Sigma-Aldrich, St. Louis, MO, USA), 100 U Penicillin G and 1000 U streptomycin, 2% fetal bovine serum (FBS; Hyclone Laboratories, Logan, UT, USA), 15 ng/mL epidermal growth factor (EGF), 15 ng/mL platelet derived growth factor (PDGF)-BB (both from R&D Systems, Minneapolis, MN, USA) and 10 ng/mL leukemia inhibitory factor (LIF; Millipore, Temecula, CA, USA). mUC-MSCs at passage numbers 12-20 were used for characterization and therapeutic treatment.

### Flow Cytometry Analysis

For mouse UC-MSC surface antigen phenotyping, cells were detached and stained with various antibodies, including anti-mouse CD2, CD4, CD5, CD11b, CD19, CD44, CD45, CD45R, TER-119, Gr-1 (eBioscience, San Diego, CA, USA), CD13, CD29, Cd49e, CD117 (BD Pharmingen, San Diego, CA, USA), CD49d, Sca-1 (BioLegend, San Diego, CA, USA), TRA-1-60, TRA-1-81, SSEA-1, and SSEA-4 (ES cell characterization kit; Millipore, Temecula, CA, USA) as previously described with minor modifications [26]. The cells were incubated for 30 min on ice with various unlabeled or specific conjugated primary antibodies and washed twice with 200 µL of cold staining buffer. Next, the cells were incubated with the respective antibody conjugated with phycoerythrin (PE) or allophycocyanin (APC) for 30 min on ice. Finally, the cells were washed twice with 200 µL of cold staining buffer and fixed with 1% paraformaldehyde until analysis with a set of FACSCalibur flow cytometer (BD Biosciences, San Jose, CA, USA).

### In Vitro Differentiation

#### Osteogenic Differentiation

To induce osteogenic differentiation, mUC-MSCs at a density of 5 × 10^3^ cells/cm^2^ were cultured in osteogenic induction medium consisting of DMEM-LG supplemented with 10% FBS, 0.1 µM dexamethasone (Sigma-Aldrich, St. Louis, MO, USA), 10 mM β-glycerol phosphate (Sigma-Aldrich, St. Louis, MO, USA), and 50 µg/mL of ascorbate-2 phosphate (Sigma-Aldrich, St. Louis, MO, USA), with medium changed every 3 days [12]. Cells were incubated for 4 weeks and then confirmed by Alizarin red staining (hydroxyapatite-associated calcium mineral deposits), von Kossa staining (CaPO_4_ crystals), and alkaline phosphatase activity, respectively [12,26].

#### Adipogenic Differentiation

To assess the adipogenic potential, mUC-MSCs at a density of 5 × 10^3^ cells/cm^2^ were cultured in the adipogenic induction medium consisting of DMEM-LG supplemented with 10% FBS, 1 µM dexamethasone, 50 µg/mL indomethacin (Sigma-Aldrich, St. Louis, MO, USA), 0.5 M 3-isobutyl-1-methylxanthine (IBMX), and 1 µg/mL insulin (Gibco, Life Technologies, Carlsbad, CA, USA) for 14 days and confirmed with oil-Red O staining (oil droplet) [23].

#### Neuroectodermal Differentiation

For neuroectodermal differentiation, mUC-MSCs at a density of 4 × 10^3^ cells/cm^2^ were cultured in an induction medium as previously described with minor modification [48]. The induction medium consisted of DMEM-HG supplemented with 2% FBS, 0.1 µM dexamethasone, 50 µg/mL ascorbic acid 2-phosphate, 50 µM indomethacin, 10 µg/mL insulin, and 0.45 mM IBMX.

### Cytochemical Staining and Immunochemistry

#### Alkaline Phosphatase Activity

For evaluation of osteogenic differentiation, the mUC-MSCs were washed twice with PBS and fixed in 4% paraformaldehyde for 10 min at room temperature. Cells were permeated with 0.2% Triton X-100 in PBS for 10 min and washed twice with PBS. Next, cells were incubated with NBT/BCIP substrate solution (Sigma-Aldrich, St. Louis, MO, USA) in the dark for 1 hr and observed under an inverted microscope (Olympus 1X70, Hamburg, Germany).

#### Oil-Red O Staining

For evaluating the generation of oil droplets, the mUC-MSCs were fixed with 10% formalin for 10 min at room temperature and washed twice with water. Oil-Red O (Sigma-Aldrich, St. Louis, MO, USA) working solution was prepared by adding 6 mL stock (0.5 g Oil-Red O in 100 mL isopropanol) to 4 mL distilled water, mixed, and filtered through Whatman #1 filter paper. Next, Oil-Red O stain was added and incubated for 1 hr at room temperature. Finally, the cells were rinsed several times with water and observed under an inverted microscope.

#### Immunocytochemistry

For evaluating the differentiation potential of UC-MSCs, the cells were fixed with 4% paraformaldehyde for 10 min at room temperature and washed twice with PBS. Cells were permeated with 0.5% Triton X-100 in PBS for 5 min and incubated in PBS with 0.2% Tween-20 (PBST) and 3% BSA for 1 hr. Next, primary antibodies, including mouse anti-nestin (1:100; Millipore, Temecula, CA, USA), rabbit anti-GFAP (1:1000; Millipore, Temecula, CA, USA), mouse anti-MAP-2 (1:500; Abcam, Cambridge, MA, USA), mouse anti-βⅢ-tubulin (1:1000; Millipore, Temecula, CA, USA), were added, respectively, and incubated overnight at 4 °C. After washing 3 times with the PBST buffer, the cells were incubated with Alexa Fluor 555-conjugated donkey anti-rabbit or mouse IgG (1:500; Life Technologies, Carlsbad, CA, USA) for 1 hr at room temperature and washed 3 times with PBST. Photographs were taken on a Zeiss LSM510 (Jena, Germany), and images were processed in the Zeiss LSM Image Examiner Version 4.2.0.121 software.

### Hematopoietic Reconstitution with mUC-MSCs

Eight- to 10-week-old male C57BL/6JNarl mice were subjected to a whole-body sub-lethal dose of 8 Gy or lethal dose of 10 Gy γ-irradiation. Five million GFP-mUC-MSCs or PBS vehicle control were administrated to mice 24 hr post-irradiation via tail vein injection as previously described with minor modifications [49,50]. Mice were sacrificed and various tissues were collected at 1, 2, 3, 4, 5, 6 weeks (sub-lethal dose), and 6 months (lethal dose) after irradiation for hematopoietic lineage differentiation of mUC-MSCs. Besides, the peripheral blood collected between 5 and 6 weeks post-irradiation were used to isolate the lineage positive cells by FACSAriaII Sorter (BD Biosciences, San Jose, CA, USA) and further analyzed by genomic DNA PCR.

### Isolation and Analysis of Lineage-positive (Lin^+^) Cells

To assess the hematopoietic differentiation *in vivo*, cardiac puncture blood collection was performed on mice that were subjected to whole body sub-lethal γ-irradiation and treated with PBS or GFP-mUC-MSCs. Following lysis of red blood cells, the samples were incubated with APC mouse lineage antibody cocktail (BD Pharmingen, San Diego, CA, USA) as summarized in the aforementioned procedure in flow cytometry analysis. Prior to cell sorting, Propidium iodide (PI) staining was used to detect dead cells in a population. Next, the PI negative and Lin^+^ cells were isolated by the FACSAriaII Sorter. The isolated Lin^+^ cells were subject to genomic DNA extraction and GFP expression was detected by PCR.

### Acute Toxic Hepatic Failure in Mice

Eight- to 10-week-old male C57BL/6JNarl mice were subjected to a single dose of 1000 mg/kg thioacetamide (TAA, Sigma-Aldrich, St. Louis, MO, USA) intraperitoneally [51]. PBS or 2 × 10^6^mUC-MSCs were administrated into mice via tail vein injection 6 hr after the induction of liver failure. Mouse survival rate was monitored daily until 14 days post-induction.

### Semiquantitative Reverse Transcriptase PCR (RT-PCR)

The total RNAs of mUC-MSCs and mouse embryonic fibroblasts (MEFs) were extracted by the TRIzol Reagent (Life Technologies, Carlsbad, CA, USA) according to the manufacturer’s protocol. Reverse transcriptase (RT) was applied in a volume of 20 μL as follows: 2 µg of total RNA and 1 µL of oligo (dT)_18_ primer were brought up to 12-µL with nuclease-free water, heated at 65 °C for 5 min, and then cooled immediately on ice. A mixture of 8 µL was prepared as: 4 µL of 5× reaction buffer, 2 µL of 10 mM dNTP mix, 1 µL of RiboLock RNase inhibitor (20 unit/μL), and 1 µL of reverse transcriptase (200 unit/μL, RevertAid M-MuLV Reverse Transcriptase; Fermentas, Lithuania). This mixture was added to the RNA sample tube and incubated for 60 min at 42 °C. The reverse transcriptase was deactivated by heating at 70 °C for 5 min. The specific primers for the target genes are listed on Table S1.

### Focal Cerebral Ischemia by Middle Cerebral Artery Occlusion (MCAo)

Ten-week-old male C57BL/6JNarl mice were subjected to MCAo. Under anesthesia with chloral hydrate (0.4 g/kg) and 5% isoflurane, ligations of the right middle cerebral artery (MCA) and right common carotid arteries (CCA) were performed as previously described, with minor modifications [52,53]. The right CCA was clamped with a non-traumatic arterial clip. With the use of a surgical microscope (Leica M651, Bensheim, Germany), the right MCA was ligated with a 10-0 nylon suture. Cortical blood flow was measured continuously with a laser-Doppler flowmeter (PF-5010, Periflux System, Perimed AB, Stockholm, Sweden) in anesthetized animals. After 2 hr of ischemia, the suture on the MCA and arterial clip on CCA were removed to allow reperfusion (ischemia/reperfusion model). During recovery from the anesthesia, the body temperature was maintained at 37 °C with a heating lamp. Brain infarct was measured by 2,3,5-triphenyltetrazolium (TTC) staining.

### Intracerebral Transplantation of UC-MSCs

Mice were subjected to transplantation with mUC-MSCs, hUC-MSCs, or PBS vehicle control 24 hr after stroke as previously described with minor modifications [26]. The cells were pre-labeled with Cell Tracker CM-DiI (Chloromethyl-benzamido-1,1’-dioctadecyl-3,3,3’3’-testramethylindo carbocyanine perchlorate; Life Technologies, Carlsbad, CA, USA) and washed 5 times with PBS before trypsinization. Under anesthesia, mice were transferred to a stereotaxic apparatus in a clean field, and the cranium was exposed through midline skin incision. Two small burr holes were made 2 mm to the right and 1 mm above and below the bregma with a dental drill. A 10-µL Hamilton syringe was inserted into the brain parenchyma following coordinates in relation to bregma: −0.5 and 1.5 mm anteroposterior, 2 mm mediolateral, and 2.5 mm dorsal. About 2 µL of the cell suspension (1 × 10^5^ cells/µL) was administrated into the peri-penumbra region over a 5-min period with an automatic microinjection pump system.

### Immunohistochemistry of Brain Tissue

Mice brains were fixed by transcardial perfusion with saline and 4% paraformaldehyde, and immersed in 4% paraformaldehyde overnight. Brain samples were dehydrated in 30% sucrose and frozen on dry ice. Ten-µm thick serial sections were cut in the coronal plane with a cryostat (Leica CM3050S, Wetzlar, Germany). Cell type-specific markers; GFAP for astrocytes, CD31 for endothelial cells, Neu-N for neuronal cells, CD11b for microglia, and nestin for neural progenitors, respectively, were used to co-stain with CM-DiI in brain sections. Histological brain sections were stained with specific antibodies: rabbit anti-GFAP (1:300; Millipore, Temecula, CA, USA), rat anti-CD31 (1:200; BD Biosciences, San Jose, CA, USA); mouse anti-Neu-N (1:100; Millipore, Temecula, CA, USA); mouse anti-nestin (1:50; Millipore, Temecula, CA, USA); and rat anti-CD11b (1:200; eBioscience, San Diego, CA, USA). The sections were left overnight at 4 °C, and incubated the following day with Alexa Fluor-conjugated donkey anti-mouse 488, chicken anti-rat 488, or donkey anti-rabbit 488 (1:500; Life Technologies, Carlsbad, CA, USA) for 3 hr at room temperature. The tissue sections were analyzed with a laser-scanning confocal microscope (Leica SP5 or Zeiss LSM510).

### Statistical Analysis

All data are expressed as mean ± SEM. The Student’s *t*-test was used to assess mean difference between the vehicle control (PBS group) and the treated group (mUC-MSCs group). *P*-value < 0.05 was considered statistically significant.

## Results

### Isolation and characterization of a novel MSC from mouse umbilical cords (mUC-MSCs)

**Figure 1:**
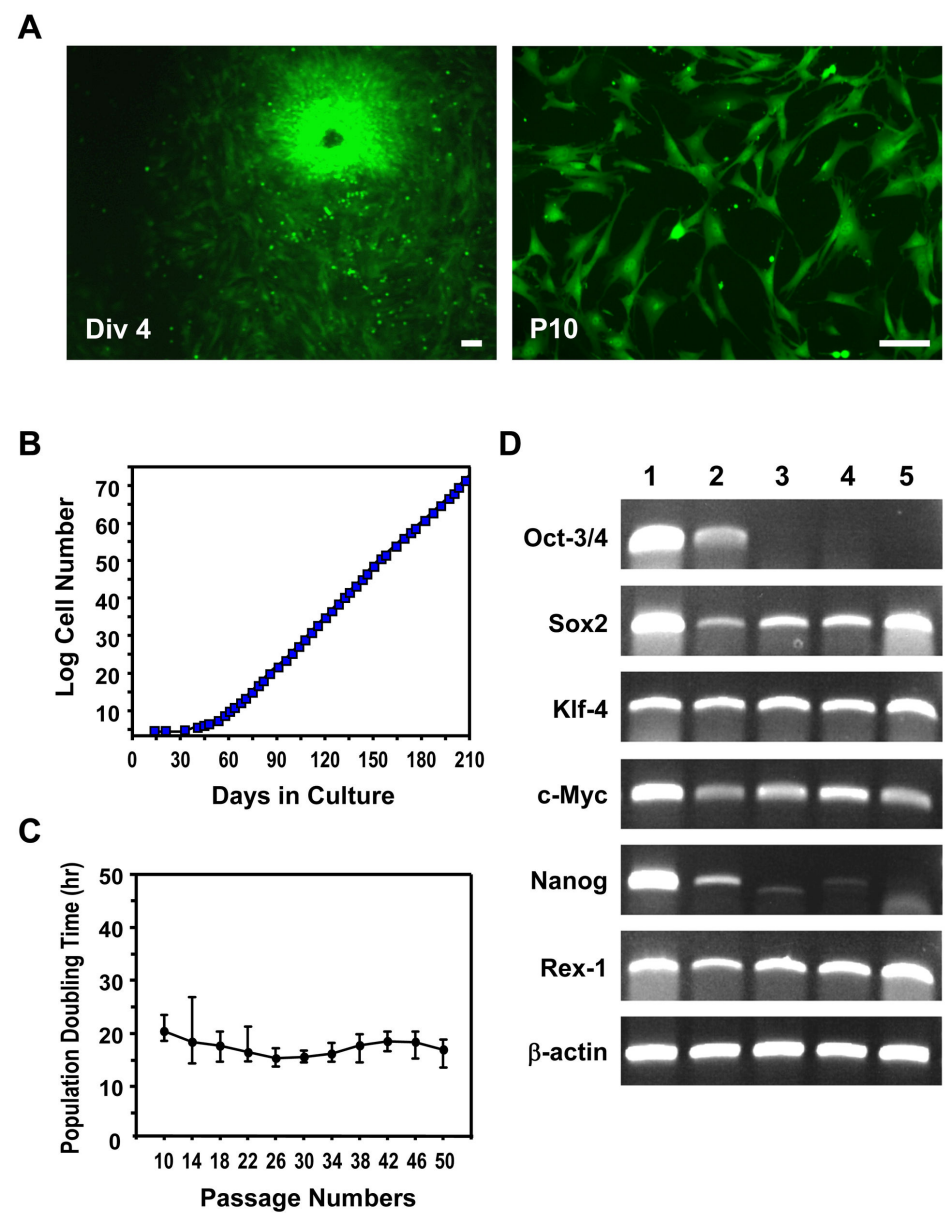
Isolation of novel MSCs from mouse umbilical cord. (A) Mouse UC-MSCs isolated from GFP transgenic mice at passage numbers 0 (Div 4) and 10 (P10). mUC-MSCs displayed the triangular, spindle-shaped, and fibroblast-like morphology at P10. (B) Growth Kinetics of mUC-MSCs. Cumulative cell number was counted at each passage. (C) The population doubling time pattern of mUC-MSCs from passage 10 to passage 50. (D) RT-PCR analysis of the pluripotency-associated genes in mUC-MSCs. Lane 1, mES cells; lane 2, germ cells from testis; lane 3, mUC-MSCs from heterogeneous populations (Mix); land 4-5, mUC-MSCs derived from different single colony. Scale bar = 50 µm.

**Figure 2:**
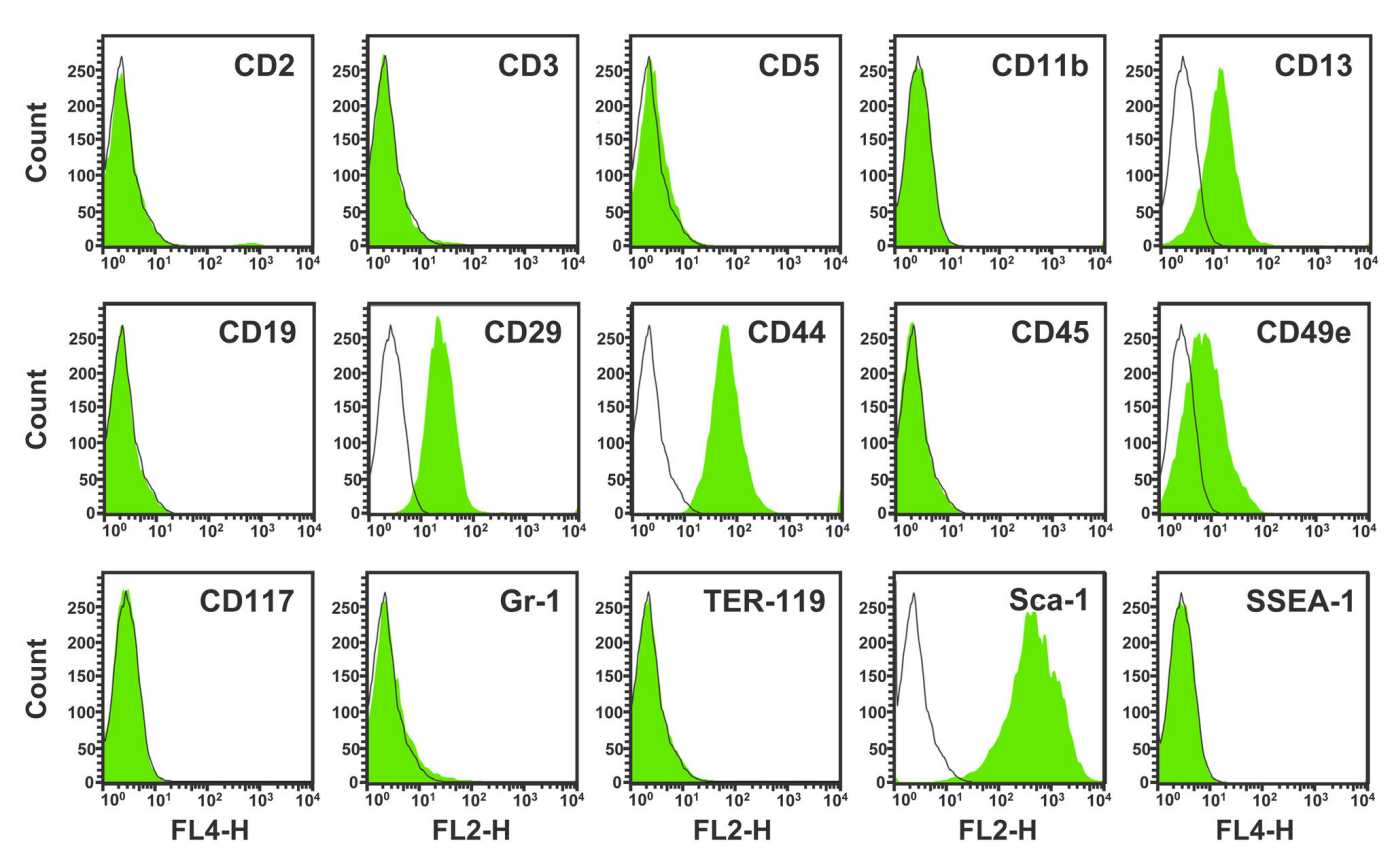
Immunophenotype antigen profile of mUC-MSCs. Cells of passage number 12–20 were labeled with PE- or APC-conjugated antibodies against the indicated antigens, and analyzed by flow cytometry. Hematopoietic cells markers, including CD2, CD3, CD5, CD11b, CD19, CD45, CD117, Gr-1, and TER-119; mesenchymal stem cells markers, including CD13, CD29, CD44, CD49e, and Sca-1; and mES cells marker, SSEA-1, were used. The respective isotype control was displayed as an open histogram (dark gray line), and specific antibody was displayed as a filled histogram (green).

A novel strain of murine MSC was successfully established and expanded from full-term umbilical cord. mUC-MSCs isolated from explanted mouse umbilical cords were expanded in culture, displaying a heterogeneous morphology with many spindle-shaped cells and small round cells, and with a high nucleus-to-cytoplasm ratio. After 10 passages, a more homogeneous population was isolated and used for studies (Figure 1A). The cells have been subjected to more than 50 passages of subculture over a period of 7 months (Figure 1B). No morphological differentiation was observed after 50 passages in cultures, and the mean population doubling time was 17.96 ± 2.22 hr for the last 10 passages (Figure 1C). Several stem cell pluripotency and self-renewal-related transcription factors Sox2, c-Myc, Rex-1, and Klf4 were expressed in mUC-MSCs, as well as, to a lesser extent, Nanog (Figure 1D). No Oct3/4 expression was observed in mUC-MSCs after 10 passages, although OCT4 was observed in freshly isolated and early passages of mUC-MSCs (Figure S1). Similarly, expression of the murine embryonic stem (mES) cell marker, SSEA-1, was also observed at early, but not late, passages. mUC-MSCs were further characterized by their immunophenotype antigen profile via flow cytometry analysis. mUC-MSCs were negative for hematopoietic markers, i.e., CD2, CD3, CD5, CD11b, CD19, CD45, CD117, Gr-1, and TER-119, but positive for mesenchymal and cell adhesion molecules, i.e., CD13, CD29, CD44, CD49e, and Sca-1 (Figure 2). We then compared surface markers among human UC-MSCs and various murine MSCs from different origins, including bone marrow, synovium, and epiphysis (Table S2). The antigen profiling data reveal that the characterization of mUC-MSCs resembles murine MSCs from various origins, but not ES cells.

### In vitro differentiation of mUC-MSCs to osteoblasts, adipocytes, and neural cells

**Figure 3:**
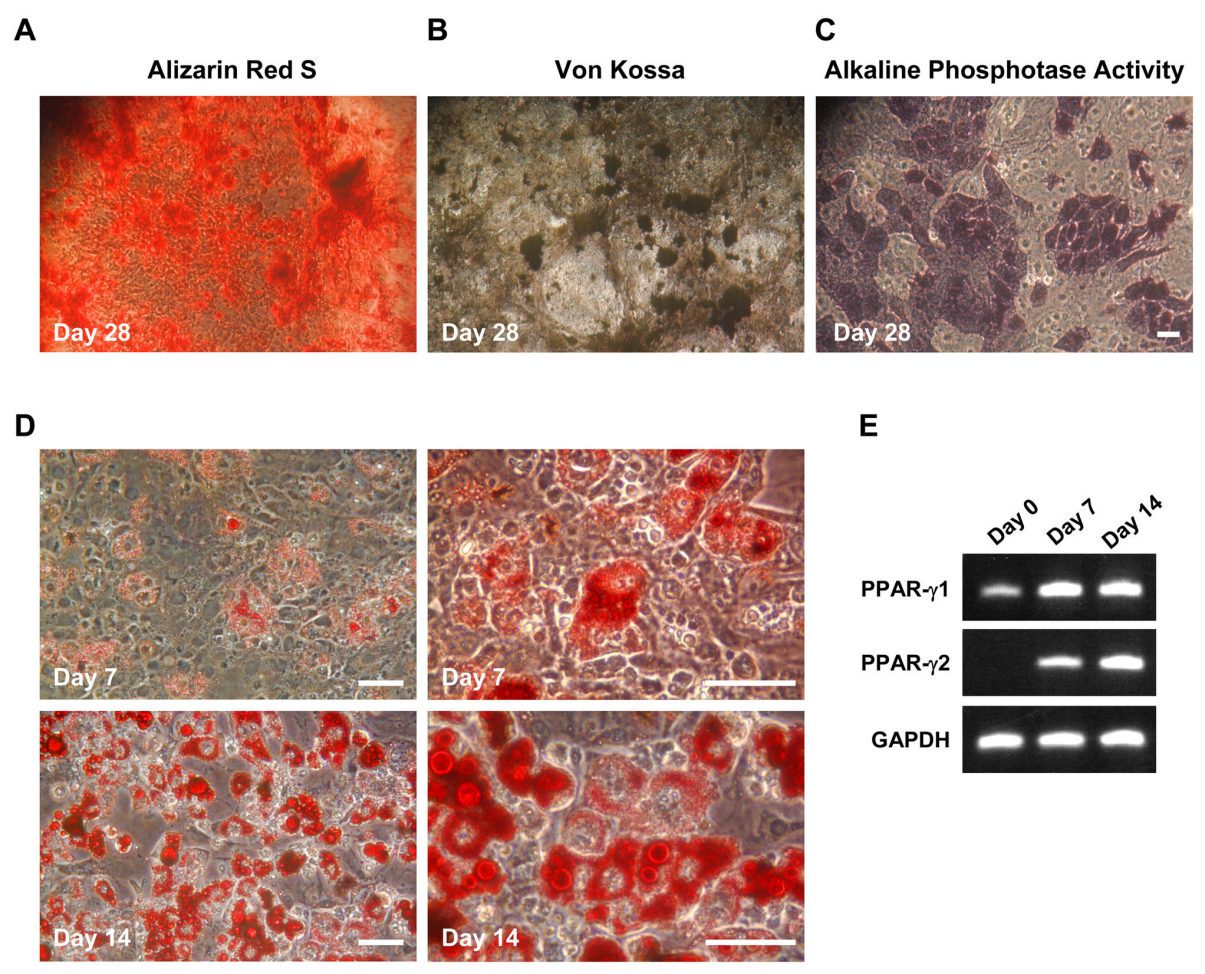
Mesodermal differentiation potential of mUC-MSCs. *Osteogenic differentiation*: Cells of passage number 12–20 were induced to form osteoblast by culturing in osteogenic induction medium for 28 days, followed by staining with Alizarin Red-S (A), von Kossa (B), and evaluated by alkaline phosphatase activity (C) for osteoblasts. *Adipogenicdifferentiation* : cells were induced to form adipocytes by culturing in induction medium for 14 days. Adipocytic differentiation is evidenced by the formation of oil droplets stained with Oil-red O. Panel D (right) displayed a higher-magnification image of cells. (E) Expression of adipocytic phenotypic markers PPAR-γ1 and PPAR-γ2 was assayed by RT-PCR. Scale bar = 50 µm.

mUC-MSCs were further evaluated by their differentiation potential, a hallmark of MSCs. To investigate the mesodermal osteogenic differentiation potential of mUC-MSCs, cells were cultured in the induction medium for 4 weeks and characterized by staining with Alizarin Red S, von Kossa, and alkaline phosphatase activity (Figure 3A–C). The differentiated cells displayed typical features for osteoblasts, including mineralized extracellular matrix, bone nodules, and high alkaline phosphatase activity *in vitro*.

To assess the mesodermal adipogenic differentiation potential of mUC-MSCs, the cells were plated and cultured in adipogenic induction medium for 14 days. Morphologic changes and lipid droplets were detectable after only 4 days, with maximal lipid accumulation at 2 weeks. The neutral lipid vacuoles were visualized by staining with Oil-red O (Figure 3D). mUC-MSCs grown under basic culture conditions expressed the transcription factor, PPAR-γ1, known to be involved in the control of adipogenic differentiation. After an induction period of 7-14 days, mUC-MSCs expressed the mature adipocyte marker, PPAR-γ2 (Figure 3E). The differentiation ability of hUC-MSCs was also investigated and compared to that of mUC-MSCs, which is displayed in supplementary data (Figure S2). It is apparent that the efficacy of differentiation of mUC-MSCs is much better than that of hUC-MSCs.

**Figure 4:**
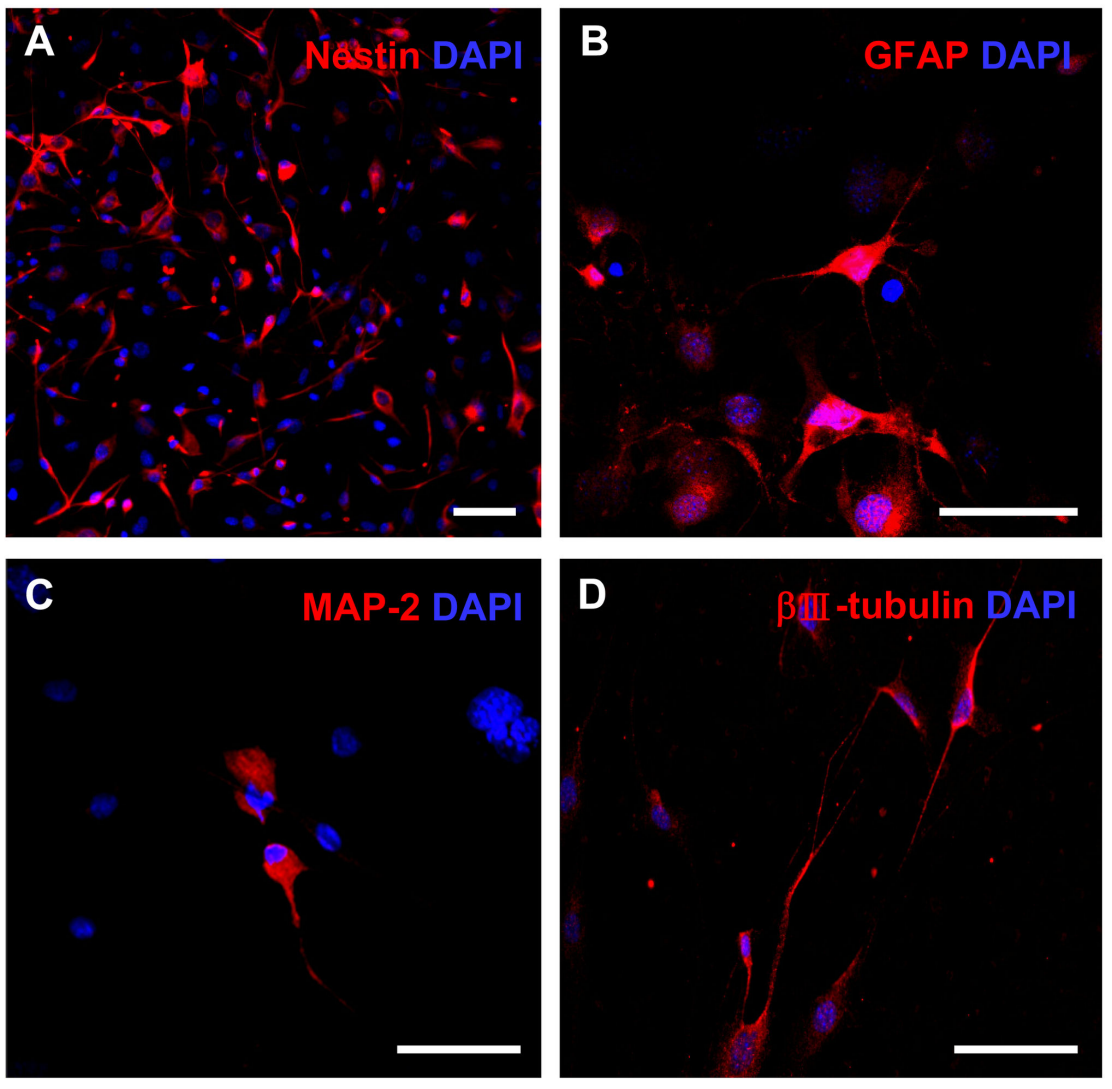
Neuroectodermal differentiation potential of mUC-MSCs. Cells of passage number 12–20 were induced to form neural cells by culturing in induction medium for 7 days. Cells were co-stained with DAPI and neural progenitor marker, nestin, at 6 hr (A); or astroglial marker, GFAP (B); and neuronal markers, including MAP 2 (C) and βⅢ-tubulin (D), at 5 days post-induction. Scale bar = 50 µm.

For neuroectodermal differentiation, the cells were cultured in neural induction medium for 1 week and analyzed by immunocytochemistry. The early changes occurring within 2-4 hr after replacement of induction medium included the rounding of cell bodies and bipolarization of thin extensions; therefore, the wide and flat induced cells transformed into a rounded shape and formed axon- or dendrite-like structures. More than half of the cells expressed nestin, a high molecular weight intermediate filament present in early neural progenitors, during the first 6 hr of differentiation (Figure 4A). Furthermore, more than 20% of differentiated cells expressed the astroglial marker GFAP at 5 days after induction (Figure 4B). Markers for developing neurons (i.e., βⅢ-tubulin) and for mature neurons (i.e., MAP 2) were observed in both cell bodies and neuronal outgrowths at later stages of differentiation (Figure 4C, D). These findings indicate that mUC-MSCs are able to differentiate into mesodermal lineage cells and also neural cells that are distinct from its origin *in vitro*. Similarly, hUC-MSCs can differentiate into neural cells (Figure S3), albeit at lower efficacy.

### Hematopoietic reconstitution with mUC-MSCs improves survival rate of γ-irradiated mice

**Figure 5:**
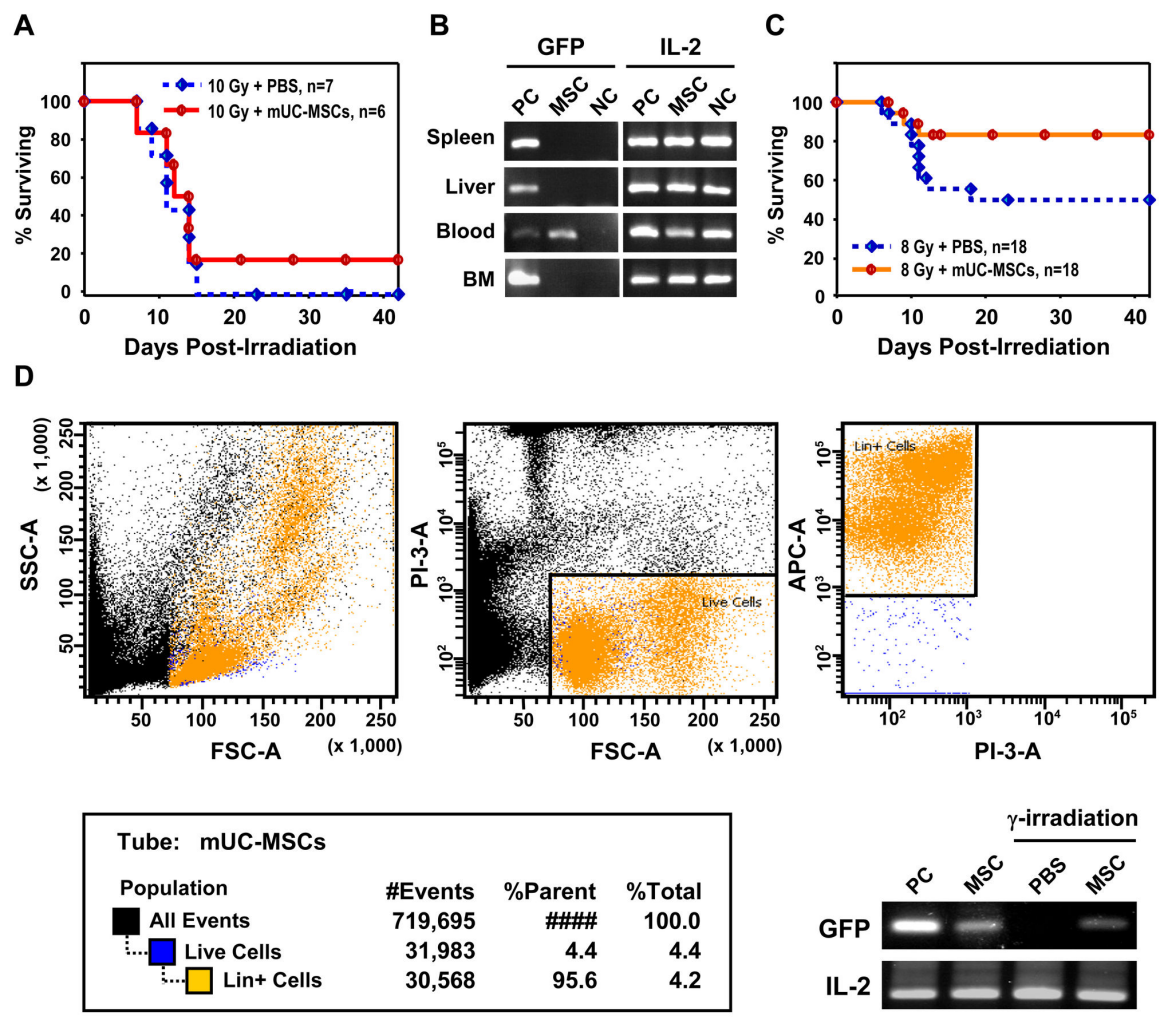
mUC-MSCs transplantation improves mice survival after hematopoietic reconstitution. (A) The C57BL/6JNarl mice were subjected to a lethal dose of 10 Gy γirradiation, and administered 5 × 10^6^ cells of GFP-mUC-MSCs (n = 6) or PBS control (n = 7) via tail vein injection 24 hr later. The survive rate of the recipient mice was analyzed. (B) The expression of GFP in liver, spleen, bone marrow (BM), and peripheral blood (PB), was analyzed 6 months post-transplantation by genomic DNA PCR. The expression of IL-2 serves as internal control. (C) The C57BL/6JNarl mice were subjected to a sub-lethal dose of 8 Gy γ-irradiation and administered with GFP-mUC-MSCs (n = 18) or PBS control (n = 18) via tail vein injection 24 hr later. (D) The hematopoietic lineage cells (Lin^+^ cells) of peripheral blood from mice (n = 6, each group) in (C) were analyzed 6 weeks after sub-lethal irradiation. The isolated Lin^+^ cells were subjected to further extraction of genomic DNA and GFP expression was analyzed by PCR. PC = positive control, the tissues or Lin^+^ cells from GFP-transgenic mice; NC = negative control, the tissues or Lin^+^ cells from wild-type C57BL/6JNarl mice; MSC = GFP-mUC-MSCs.

It has been reported that the MSCs isolated from adult mouse bone marrow are capable of differentiating into hematopoietic lineage cells [47]. To evaluate the ability of mUC-MSCs in hematopoietic reconstitution *in vivo*, a model of mouse bone marrow transplantation was applied. C57BL/6J mice were subjected to a whole-body lethal dose of 10 Gy γ-irradiation, followed by administration of 5 × 10^6^GFP-mUC-MSCs or PBS vehicle control at 1 day after irradiation. Only 1 out of 6 lethally irradiated-mice survived after transplantation of mUC-MSCs (Figure 5A). Tissues involved in hematopoiesis, including spleen, liver, bone marrows, and peripheral blood, were further analyzed after reconstitution. Although, the engrafted GFP-mUC-MSCs could be tracked by GFP fluorescence, a consistent and significant decrease in GFP expression by MSCs after isolation from GFP transgenic rodent has been reported [54], a result also observed in our preparations. To eliminate undetectable expression of GFP from gene silencing, tracing of GFP-mUC-MSCs was assessed by genomic DNA PCR. Notably, the GFP gene is still retained in circulating peripheral blood of surviving mice at 6 months after engraftment of mUC-MSCs (Figure 5B). In order to reduce the mortality rate, a whole-body sub-lethal dose of 8 Gy was performed. After transplantation of mUC-MSCs, the survival rate increased (15/18, 83.3%) compared to the PBS-treated group (9/18, 50%; Figure 5C). The Lin^+^ cells in the circulating peripheral blood were harvested from PBS vehicle control or GFP-mUC-MSCs-treated group by flow cytometric cell sorting at 6 weeks post-transplantation. Genomic DNA PCR analysis further revealed that the GFP-mUC-MSCs subsisted in hematopoietic Lin^+^ cells in mUC-MSCs–treated mice (Figure 5D). This finding suggests that mUC-MSCs possess the capability to differentiate into hematopoietic cells *in vivo*.

### Allograft of mUC-MSCs significantly reduces infarct volume and reveals multipotent differentiation potential in stroke model

**Figure 6:**
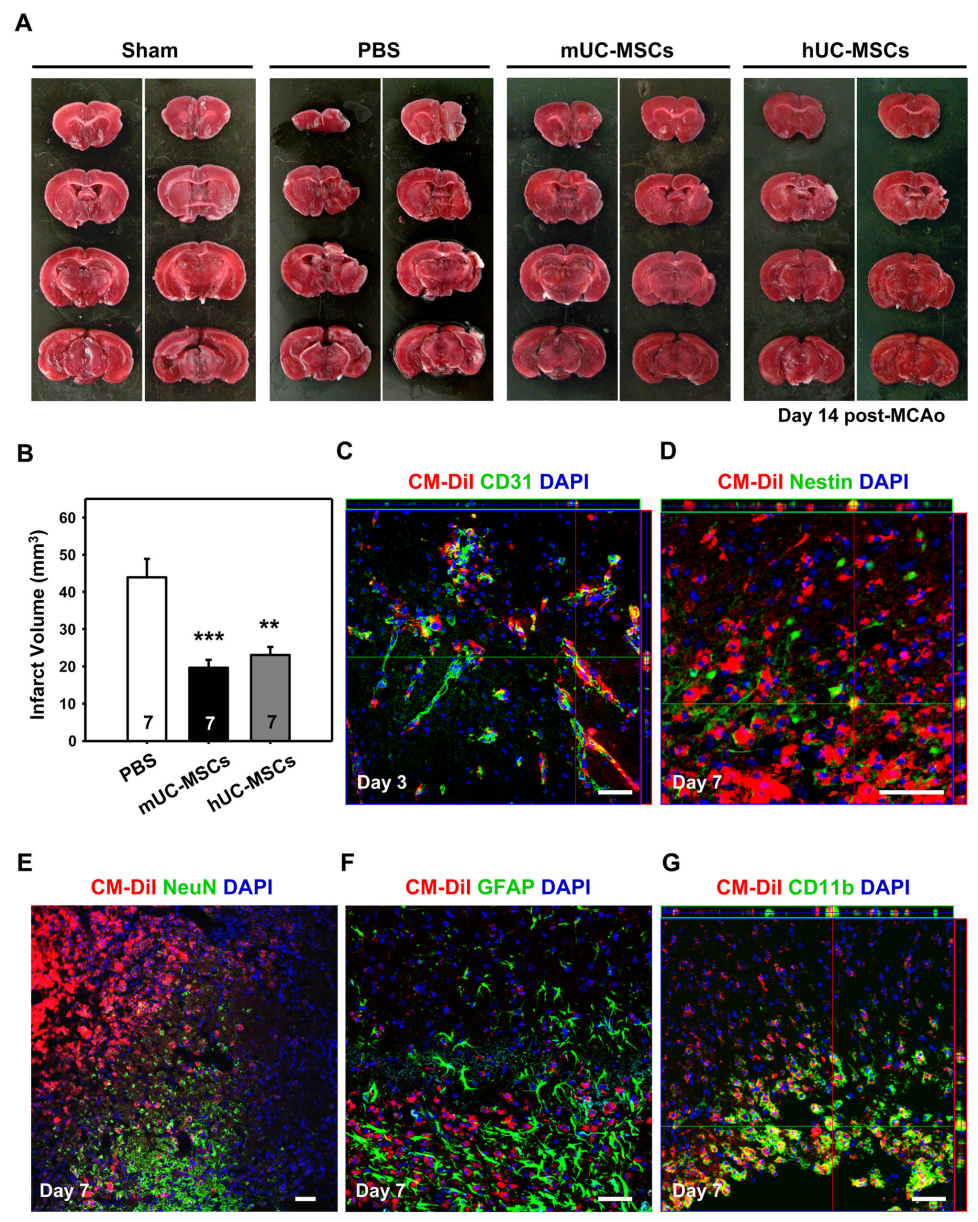
mUC-MSCs transplantation attenuates ischemic brain injury. (A) The C57BL/6JNarl mice were administrated 4 × 10^5^ cells of mUC-MSCs, hUC-MSCs, or PBS control at 1 day after a 120-min MCAo. Infarct volume was evaluated 14 days later. (B) Quantification of infarct volume in mice with mUC-MSCs (n = 7), hUC-MSCs (n = 7), or PBS control (n = 7) treatment. For assessment of differentiation potential of mUC-MSCs *in vivo*, mUC-MSCs were pre-labeled with Cell Tracker CM-DiI fluorescent dye and co-stained with specific cellular markers, including endothelial cell marker, CD31, at day 3 (C); or neural progenitor marker, nestin; neuron marker, NeuN; astroglial marker, GFAP; and macrophage/microglia marker, CD11b at day 7 (D–G) after MCAo. Data shown here are displayed as the mean ± SEM. **P < 0.01, ***P < 0.001 versus PBS control.

To investigate the potential of mUC-MSCs in the treatment of neurological disorders, a focal ischemia model by right MCA occlusion was adopted. Either PBS or 4 × 10^5^ mUC-MSCs were administrated 24 hr post-MCAo, infarct volume was significantly reduced (*P* < 0.001) in stroke mice treated with either mUC-MSCs (19.61 ± 5.67 mm^3^, n = 7), or hUC-MSCs (23.07 ± 5.77 mm^3^, n = 7) compared to the PBS treated-group (43.94 ± 13.06 mm^3^, n = 7; Figure 6A, B). In order to investigate the differentiation capability of mUC-MSCs in ischemic brain, mUC-MSCs were pre-labeled with Cell Tracker CM-DiI before transplantation, and cell fate was evaluated after cerebral ischemia by fluorescence immunostaining assays. In the penumbra of mUC-MSCs-treated ischemic brains, CM-DiI-labeled cells were also co-stained with the vascular phenotype marker, CD31, about the perivascular and endothelial regions (Figure 6C). CM-DiI-labeled cells were stained positively for the neural progenitor cell marker, nestin (Figure 6D), but not neuronal marker, NeuN (Figure 6E) nor the astroglial marker, GFAP (Figure 6F). Furthermore, CM-DiI-labeled cells were also stained positively for a microglia marker, CD11b (Figure 6G). These results indicate that allogeneic transplantation of mUC-MSCs could attenuate ischemic brain insult and revealed multipotent differentiation potential *in vivo*.

### mUC-MSCs increase mice survival rate after TAA-induced acute hepatic failure

**Figure 7:**
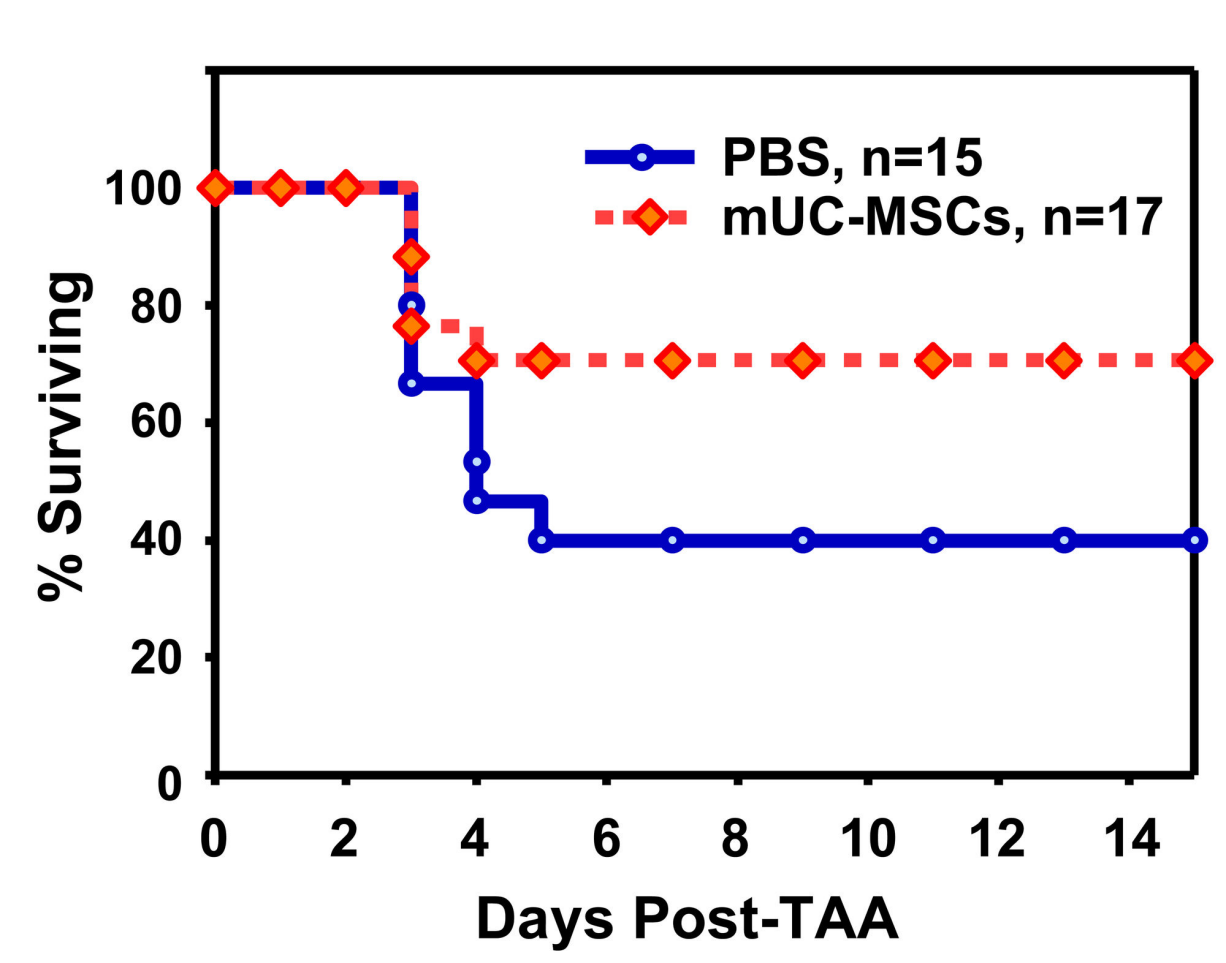
mUC-MSCs transplantation improves mice survival after TAA-induced acute hepatic failure. Male C57BL/6JNarl mice were administrated TAA (1000 mg/kg intraperitoneal injection) 6 hr prior to receiving 2 × 10^6^ cells of mUC-MSCs (n = 17) or PBS control (n = 15) via tail vein injection, and the survival rate was analyzed.

To evaluate the therapeutic effect of mUC-MSCs, mice were intraperitoneally administrated a single dose of TAA (1000 mg/kg) to induce the acute hepatic failure, followed by supplementation with PBS or 2 ± 10^6^ mUC-MSCs by intravenous administration at 6 hr after TAA injection. Approximately 60% of mice died in the PBS group between 48–120 hr in the TAA-induced acute hepatic failure model. A significant improvement in the survival rate was observed in mUC-MSCs-treated group (70.71 ± 0.41%, n = 17) compared to the PBS group (39.29 ± 6.19%, n = 15) until 14 days after transplantation (Figure 7).

## Discussion

Although encouraging initial results have been observed in the application of hUC-MSCs for the treatment of various diseases, advances in therapeutic efficacy have been hampered by the lack of detailed understanding of its cellular and molecular mechanisms. Xenogeneic immune responses further complicated the host cell responses, despite the hypoimmunogenic nature of hUC-MSCs [5–9,40,41]. We set out to isolate mouse UC-MSCs to address these deficiencies. Furthermore, there are several advantages to use mouse UC-MSCs over hUC-MSCs, including fewer ethical concerns, relatively easy sampling and gene manipulation, and allogeneic transplantation mimicry. In the present study, a novel strain of MSCs from mouse umbilical cord was isolated and was capable of sustained self-renewal for up to 7 months (over 50 passages) without overt changes in the morphology and doubling time. Despite the observations that morphology and surface marker of mUC-MSCs are similar to hUC-MSCs (Table S2), the culturing environments are quite distinct; the former is grown on a fibronectin-coated surface and supplement with LIF and growth factors, including EGF and PDGF-BB. Nonetheless, mUC-MSCs can also be differentiated into adipocytes, osteoblasts, and neural cells as hUC-MSCs can, and at a substantially higher degree of differentiation (Table S3). In 1999, Bjornson et al. first demonstrated that ROSA26-derived neural stem cells could differentiate into a variety of blood cell types, including myeloid and lymphoid cells [49]. MSCs isolated from murine skeletal muscle [50] and bone marrow [47] also possessed hematopoietic potential, however the hematopoietic differentiation potential regarding other origins of MSCs is lacking. In this study, the mUC-MSCs stained positively for a major hematopoietic lineage cocktail antibody, including CD3, CD11b, CD45R/B220, Ly-76, Ly-6G, and Ly-6C (Figure 4D), after transplantation into sub-lethally irradiated mice. These mice demonstrating fetal-type mUC-MSCs possessed a very broad differentiation repertoire in addition to regulation and enhancement of hematopoiesis.

Major transcription factors, such as Sox2, c-Myc, Rex-1, and Klf4, that participate in stem cell pluripotency and self-renewal are expressed in mUC-MSCs. Interestingly, apart from Sox2, c-Myc and Klf4, freshly isolated mUC-MSCs also expressed OCT4 (Figure S1), a key transcription factor for pluripotency of ES cells, suggesting that pluripotent cells might exist in mouse umbilical cord. In consideration of the loss of OCT4 expression following long-term passaging, alternative methods, such as collagenase digestion plus immunodepletion, could be applied; then, several exhaustive analyses, such as reproductive cloning and gene targeting in stem cells followed by subsequent blastocyst injection, could be performed with mUC-MSCs in stem cell biology research. The lack of OCT4 may also eliminate the possibility of teratoma formation (Figure S4).

In addition to characterization of mUC-MSCs, we also investigated its therapeutic potential in two disease models, focal ischemic stroke and acute hepatic failure. In accord with hUC-MSCs, transplanted mUC-MSCs reduced infarct volume and were capable of differentiating into various types of cells in ischemic brain, e.g., neural progenitor cells, endothelial cells, and macrophages. However, the *in vitro* differentiation capability of mUC-MSCs is superior to that of hUC-MSCs (Table S3). By contrast, unlike hUC-MSCs, mUC-MSCs do not express certain cytokines, e.g., brain-derived neurotrophic factor (BDNF) and hepatocyte growth factor (HGF) (Table S4). Therefore, the differential mechanism resulting in the beneficial effect of hUC-MSCs versus mUC-MSCs remains to be identified. The allograft of mUC-MSCs used to dissect the molecular mechanism of recovery after ischemic stroke is an ongoing project in our laboratory. In the acute hepatic failure study, TAA, a potent hepatotoxin leading to centrilobular necrosis and nephrotoxic damage following acute administration, was used instead of the more commonly used hepatotoxic agent, CCl_4_, to induce acute hepatic failure since the CCl_4_-induced hepatic necrosis presents a pattern different from that of human liver damage. Similar to hUC-MSCs treatment, mUC-MSCs transplantation significantly increases the survival rate in acute hepatic failure mice [55]. Although several studies have indicated that the hepatogenic differentiation potential of MSCs contributes in rescuing mice with acute hepatic failure [56–58], it is generally agreed that the therapeutic effects of MSCs in attenuating liver injury appear to result from trophic support [55,59,60]. Interestingly, semi-quantitative RT-PCR revealed that cultured mUC-MSCs express mRNA of VEGF, FGF2, Ang2, PDGFβ, IL-6, SCF, Cxcl12, TGFβ, iNOS, and GDNF, but not IL-10, HGF, and BDNF, which are expressed by human MSCs [33,59,61–64] (Table S4). The modality with which different expression profiling in the cytokine-related genes of hUC-MSCs versus mUC-MSCs contributed to the comparable beneficial effect of the cells on acute hepatic failure deserves further investigation.

In conclusion, we have isolated and expanded a novel strain of MSCs from murine umbilical cord, which exhibits similar stem cell characteristics and therapeutic potential as hUC-MSCs, yet with substantial differences in the degree, capability, and fate of differentiation, as well as distinct cytokine expression profiles. Therefore, allograft of mUC-MSCs to various murine diseases models might shed more light on the precise cellular and molecular mechanisms underlying the beneficial effects of stem cell therapy.

## Supporting Information

Figure S1 OCT4 protein expressed in early passages of mUC-MSCs. mUC-MSCs were recognized by mesenchymal stem cell marker, Sca-1, and co-localized with pluripotent marker, OCT4.
(TIF)

Figure S2 Mesodermal differentiation potential of hUC-MSCs. hUC-MSCs of passage number 5-10 can differentiate into osteoblasts, as displayed by nodules (A), Alizarin Red-S (B), von Kossa (C), osteopontin expression (D); as well as adipocytes by lipid vacuoles: (E, F), Oil-red O (G), PPAR-γ2 expression (H). Scale bar = 50 µm.
(TIF)

Figure S3 Neuroectodermal differentiation potential of hUC-MSCs. Neural differentiated hUC-MSCs were identified by morphology (A), βⅢ-tubulin (B), MAP-2 (C), and GFAP staining (D). Scale bar = 50 µm.
(TIF)

Figure S4 No tumorigenicity in mUC-MSCs. Nude mice were administered 1 × 10^6^ cells of mUC-MSCs (red arrow) or colon-cancer cell line (blue arrow) for 2 months by subcutaneous injection. No tumor formation was observed in the mUC-MSCs treated side of the mouse.
(TIF)

Table S1 The sequences for the primers of RT-PCR. (DOCX)

Table S2 Comparison of surface markers in mouse and human-derived MSCs. (DOCX)

Table S3 Comparison of differentiation capacity of mouse and human-derived MSCs. (DOCX)

Table S4 Comparison of cytokine and chemokine profile from mouse UC-MSCs and human-derived MSCs. (DOCX)
