# Supplementary figures and images for: Isolation and Characterization of a Novel Strain of Mesenchymal Stem Cells from Mouse Umbilical Cord: Potential Application in Cell-Based Therapy
Source: PLoS One. 2013 Aug 26;8(8):e74478. doi: 10.1371/journal.pone.0074478 (PMC3753309; doi:10.1371/journal.pone.0074478)

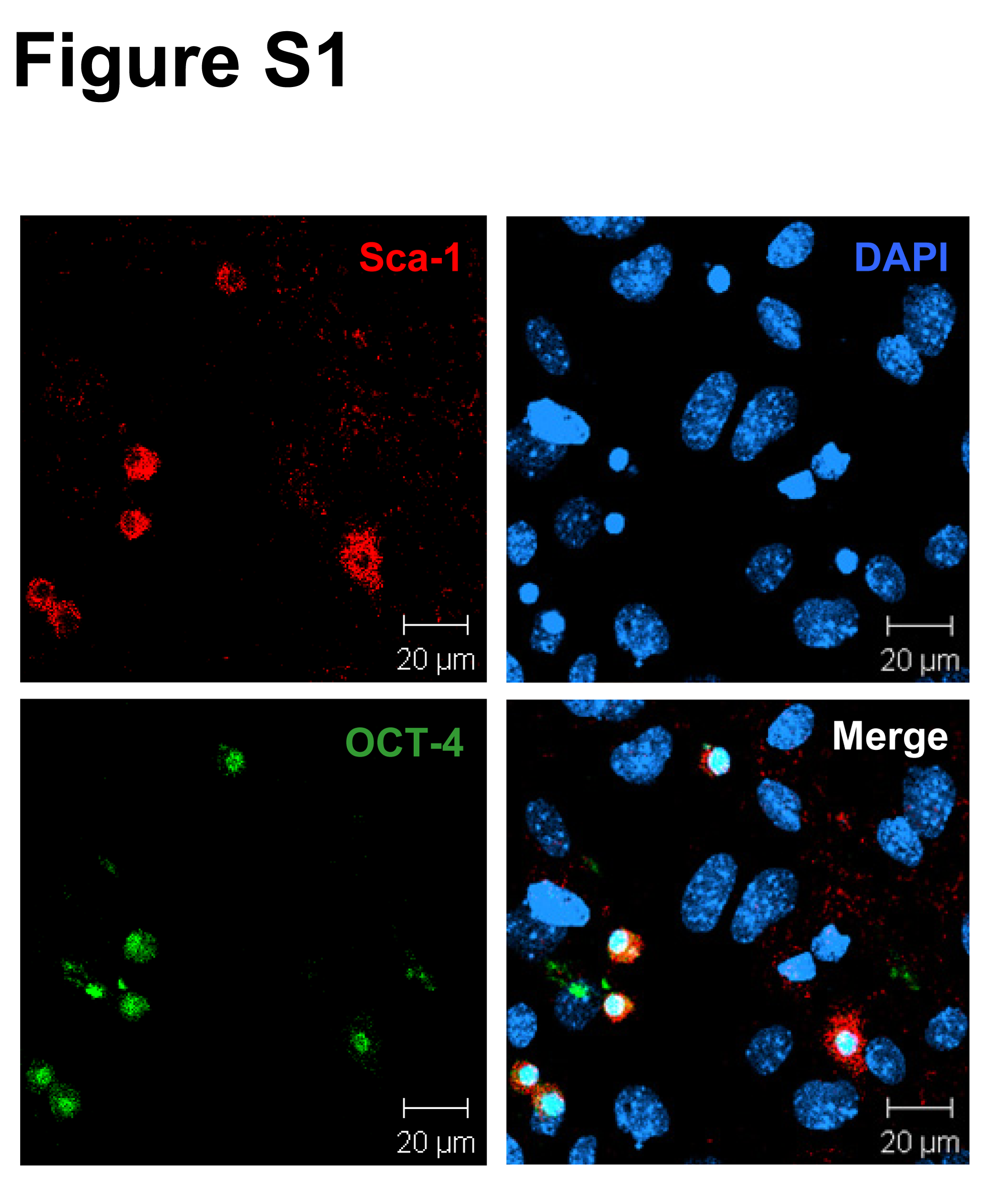

Supplement: Figure S1 — mUC-MSCs were recognized by mesenchymal stem cell marker, Sca-1, and co-localized with pluripotent marker, OCT4. (TIF) [file pone.0074478.s001.tif]

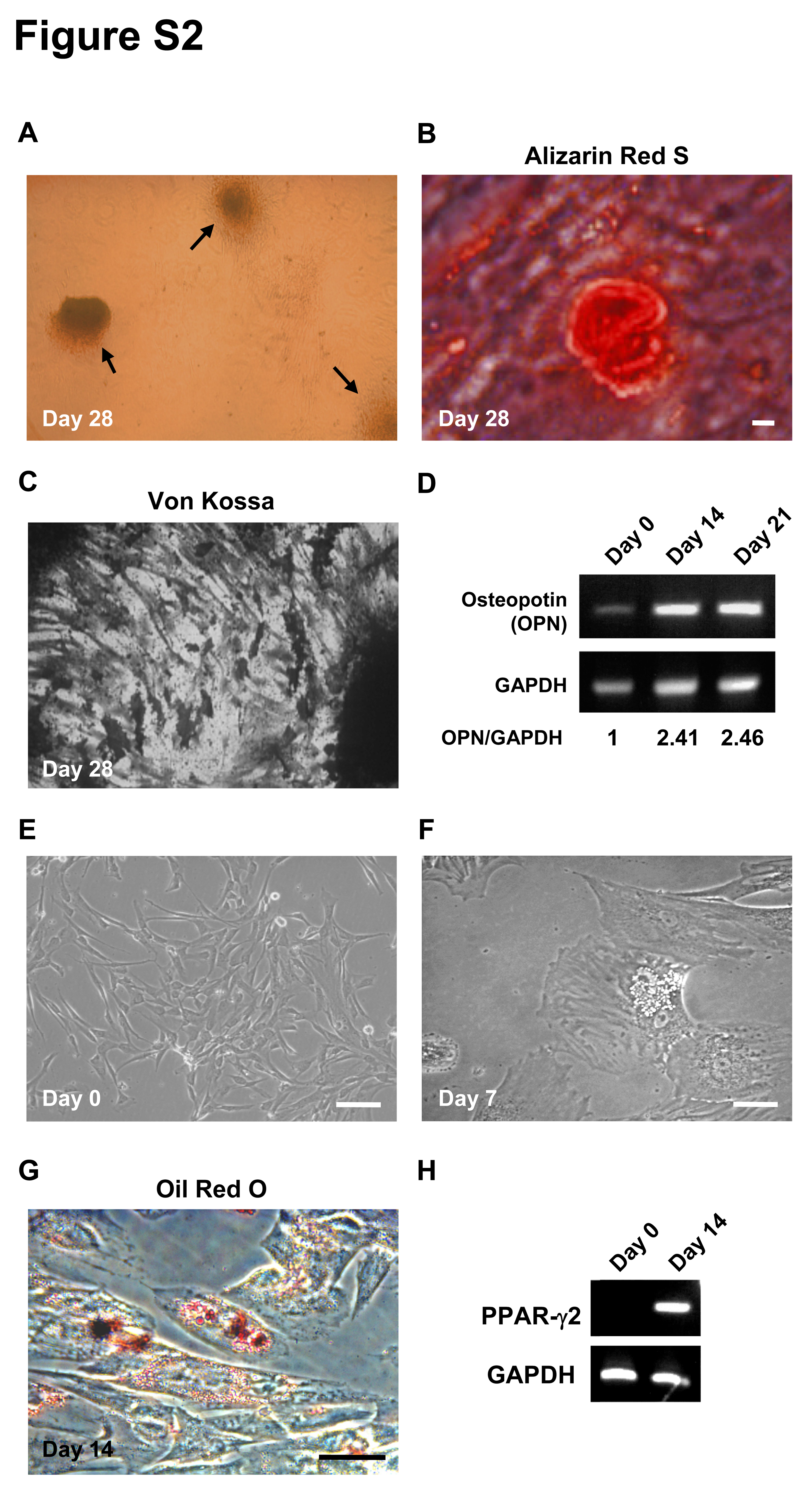

Supplement: Figure S2 — hUC-MSCs of passage number 5-10 can differentiate into osteoblasts, as displayed by nodules (A), Alizarin Red-S (B), von Kossa (C), osteopontin expression (D); as well as adipocytes by lipid vacuoles: (E, F), Oil-red O (G), PPAR-γ2 expression (H). Scale bar = 50 µm. (TIF) [file pone.0074478.s002.tif]

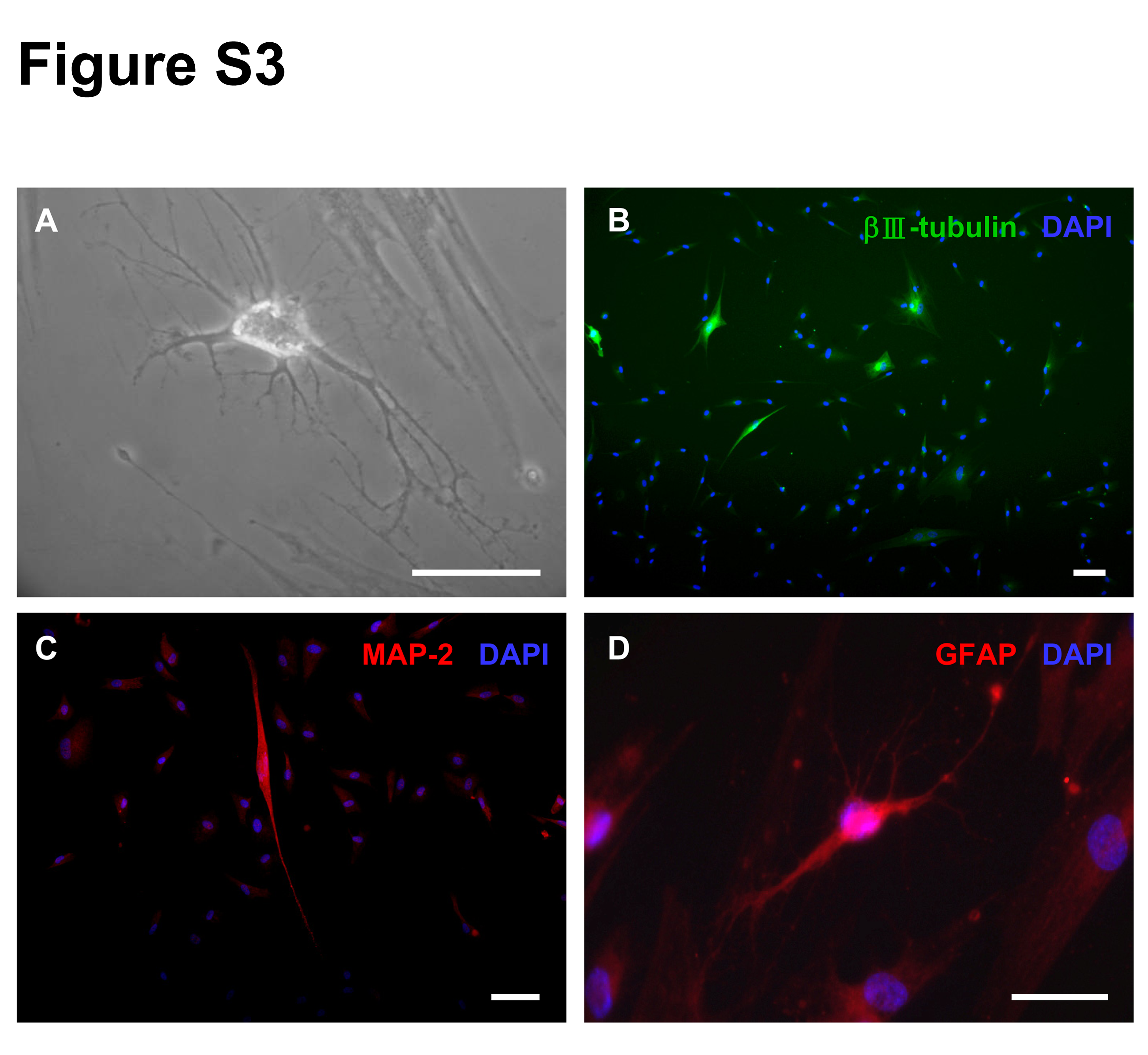

Supplement: Figure S3 — Neural differentiated hUC-MSCs were identified by morphology (A), βⅢ-tubulin (B), MAP-2 (C), and GFAP staining (D). Scale bar = 50 µm. (TIF) [file pone.0074478.s003.tif]

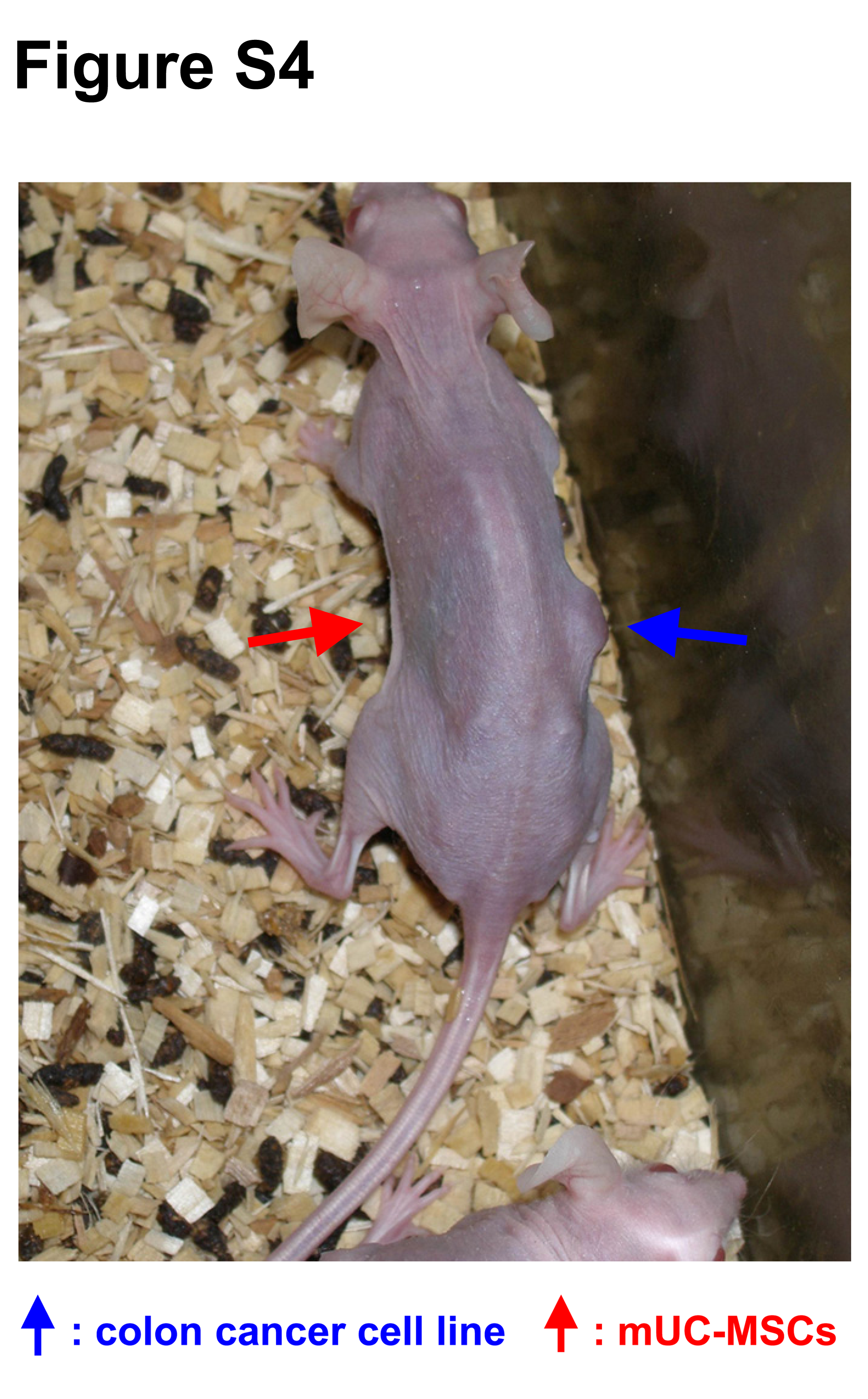

Supplement: Figure S4 — Nude mice were administered 1 × 10^6^ cells of mUC-MSCs (red arrow) or colon-cancer cell line (blue arrow) for 2 months by subcutaneous injection. No tumor formation was observed in the mUC-MSCs treated side of the mouse. (TIF) [file pone.0074478.s004.tif]
