# Supplementary material for: Isolation and Characterization of a Novel Strain of Mesenchymal Stem Cells from Mouse Umbilical Cord: Potential Application in Cell-Based Therapy
Source: PLoS One. 2013 Aug 26;8(8):e74478. doi: 10.1371/journal.pone.0074478 (PMC3753309; doi:10.1371/journal.pone.0074478)
Supplement: Table S1 — (DOCX) [file pone.0074478.s005.docx]

**Table S1.** The sequences for the primers of RT-PCR

| Gene | Primer sequence | Product length |
| --- | --- | --- |
| Oct-3/4 | F: 5’-TCTTTCCACCAGGCCCCCGGCTC-3’ | 225 bp |
|  | R: 5’-TGCGGGCGGACATGGGGAGATCC-3’ |  |
| Rex-1 | F: 5’-ACGAGTGGCAGTTTCTTCTTGGGA-3’ | 293 bp |
|  | R:5’-TATGACTCACTTCCAGGGGGCACT-3’ |  |
| Sox2 | F: 5’-TAGAGCTAGACTCCGGGCGATGA-3’ | 297 bp |
|  | R: 5’-TTGCCTTAAACAAGACCACGAAA-3’ |  |
| Nanog | F: 5’-AGGGTCTGCTACTGAGATGCTCTG-3’ | 364 bp |
|  | R: 5’-CAACCACTGGTTTTTCTGCCACCG-3’ |  |
| Klf4 | F: 5’-GCGAACTCACACAGGCGAGAAACC-3’ | 709 bp |
|  | R: 5’-TCGCTTCCTCTTCCTCCGACACA-3’ |  |
| c-Myc | F: 5’-TGACCTAACTCGAGGAGGAGCTGGAATC-3’ | 170 bp |
|  | R:5’-AAGTTTGAGGCAGTTAAAATTATGGCTGAAGC-3’ |  |
| PPAR-γ1 | F: 5’-TTCTGACAGGACTGTGTGACAG-3’ | 354 bp |
|  | R: 5’-ATAAGGTGGAGATGCAGGTTC-3’ |  |
| PPAR-γ2 | F: 5’-GCTGTTATGGGTGAAACTCTG-3’ | 350 bp |
|  | R: 5’-ATAAGGTGGAGATGCAGGTTC-3’ |  |
| GDNF | F: 5’-GATGGGTCTCCTGGATGGGAT-3’ | 737 bp |
|  | R: 5’-CAGATACATCCACACCGTTTAGCG-3’ |  |
| IL-6 | F: 5’-TGGAGTCACAGAAGGAGTGGCTAAG-3’ | 155 bp |
|  | R: 5’-TCTGACCACAGTGAGGAATGTCCAC-3’ |  |
| CXCL12 | F: 5’-AGCCTGAGCTACCGATGCCCCT-3’ | 336 bp |
|  | R: 5’-GGATGTCAGCCTTCCTCGGGG-3’ |  |
| VEGF | F: 5’-TTACTGCTGTACCTCCACC-3’ | 189 bp |
|  | R: 5’-ACAGGACGGCTTGAAGATG-3’ |  |
| PDGFβ | F: 5’-AAGCACACGCATGACAAG-3’ | 109 bp |
|  | R: 5’-GGGGCAATACAGCAAATAC-3’ |  |
| HGF | F: 5’-CGGATAGGAGCCACAAGGATCT-3’ | 510 bp |
|  | R: 5’-GCTGCCTCCTTTACCAATGATG-3’ |  |
| FGF2 | F: 5’-AACGGCGGCTTCTTCCTGC-3’ | 306 bp |
|  | R: 5’-GATCGGAGTTTATACTGCCCAGTTC-3' |  |
| BDNF | F: 5’-GACACTGAGTCTCCAGGACAGCA-3’ | 443 bp |
|  | R: 5’-CGCCGAACCCTCATAGACAT-3’ |  |
| SCF | F: 5’-CCTTAGGAATGACAGCAGTAGC-3’ | 113 bp |
|  | R: 5’-AGCCAATTACAAGCGAAATGAG-3’ |  |
| Ang2 | F: 5’-CTGTGCGGAAATCTTCAAGTC-3’ | 146 bp |
|  | R: 5’-TGCCATCTTCTCGGTGTTG-3’ |  |
| TGF-β | F: 5’-TGGTGGACCGCAACAAC-3’ | 404 bp |
|  | R: 5’-AGCCACTCAGGCGTATCAG-3’ |  |
| GAPDH | F: 5’ -ACTTTgTCAAgCTCATTTCC-3’ | 267 bp |
|  | R: 5’ –TGCAGCGAACTTTATTGATG-3’ |  |
| β-actin | F: 5’-CTCTTCCAGCCTTCCTTCC-3’ | 313 bp |
|  | R: 5-ACTCGTCATACTCCTGCTTGC-3’ |  |
| GFP | F: 5’-AAGTTCATCTGCACCACCG-3’ | 173 bp |
| (for GT) | R: 5’-TCCTTGAAGAAGATGGTGCG-3’ |  |
| IL-2 | F: 5’-CTAGGCCACAGAATTGAAAGATCT-3’ | 324 bp |
| (for GT) | R: 5’-GTAGGTGGAAATTCTAGCATCATCC-3’ |  |

Abbreviation: GT=Genotyping
