# Supplementary material for: Isolation and Characterization of a Novel Strain of Mesenchymal Stem Cells from Mouse Umbilical Cord: Potential Application in Cell-Based Therapy
Source: PLoS One. 2013 Aug 26;8(8):e74478. doi: 10.1371/journal.pone.0074478 (PMC3753309; doi:10.1371/journal.pone.0074478)
Supplement: Table S2 — (DOCX) [file pone.0074478.s006.docx]

**Table S2.** Comparison of surface markers from mouse and human-derived MSCs

|  | Mouse MSCs | | | | Human MSCs |
| --- | --- | --- | --- | --- | --- |
|  | Umbilical cord (UC-MSCs) | Bone marrow  (BM-MSCs) | Synovium  (SMCs) | Epiphysis  (EMSCs) | Umbilical cord  (UC-MSCs) |
| Hematopoietic markers |  |  |  |  |  |
| CD2 | - | ND | ND | ND | ND |
| CD3 | - | ND | ND | ND | - |
| CD5 | - | ND | ND | ND | ND |
| CD11b | - | - | ND | - | ND |
| CD19 | - | ND | ND | ND | ND |
| CD34 | ND | - | - | - | - |
| CD45 | - | - | - | - | - |
| CD45R | - | ND | ND | ND | ND |
| CD117 | - | - | - | - | - |
| Gr-1 | - | ND | ND | ND | ND |
| TER-119 | - | ND | ND | ND | ND |
| Mesenchymal markers |  |  |  |  |  |
| CD13 | + | + | ND | ND | + |
| CD73 | ND | + | ND | + | + |
| CD90 | ND | +/- | ND | ND | + |
| CD105 | ND | + | ND | + | + |
| Sca-1 | + | + | + | + | ND |
| Cell adhesion molecules |  |  |  |  |  |
| CD29 | + | + | + | + | + |
| CD44 | + | + | + | + | + |
| CD49d | + | ND | ND | ND | - |
| CD49e | + | + | ND | ND | ND |
| CD106 | ND | +/- | + | Low | ND |
| CD166 | ND | - | ND | Low | + |
| ES cell markers |  |  |  |  |  |
| SSEA-1 | - | +/- | ND | ND | ND |
| TRA-1-60 | - | ND | ND | ND | ND |
| TRA-1-81 | - | ND | ND | ND | ND |
| SSEA-4 | - | ND | ND | ND | + |
| References |  | [47, 60, 61] | [63] | [64] | [26] |

Abbreviation: ND= not determined; +, positive; -, negative
