# Supplementary material for: Isolation and Characterization of a Novel Strain of Mesenchymal Stem Cells from Mouse Umbilical Cord: Potential Application in Cell-Based Therapy
Source: PLoS One. 2013 Aug 26;8(8):e74478. doi: 10.1371/journal.pone.0074478 (PMC3753309; doi:10.1371/journal.pone.0074478)
Supplement: Table S3 — (DOCX) [file pone.0074478.s007.docx]

**Table S3.** Comparison of differentiation capacity from mouse and human-derived MSCs

|  | mUC-MSCs | hUC-MSCs |
| --- | --- | --- |
| Differentiation *in vitro* |  |  |
| Osteoblasts | + + + | + |
| Adipocytes | + + + + | ++ |
| Neural precursors | + + + | + |
| Neurons | + | + |
| Astrocytes | + + | + |
| Differentiation *in vivo* (stroke) |  |  |
| Neural precursors | + | ND [26]  [26] |
| Neurons | Nd | + [26] |
| Astrocytes | Nd | + [26] |
| Endothelial cells | + | + [26] |
| Macrophage/Microglia | + | + [26] |

Abbreviation: Nd= not detectable; ND= not determined.

Differentiation capacity was evaluated by cytochemical staining or immunostaining.

+, 1%~25%; + +, 26%~50%; + + +, 51%~75%; + + + +, > 75%
