# Supplementary material for: Isolation and Characterization of a Novel Strain of Mesenchymal Stem Cells from Mouse Umbilical Cord: Potential Application in Cell-Based Therapy
Source: PLoS One. 2013 Aug 26;8(8):e74478. doi: 10.1371/journal.pone.0074478 (PMC3753309; doi:10.1371/journal.pone.0074478)
Supplement: Table S4 — (DOCX) [file pone.0074478.s008.docx]

**Table S4.** Comparison of cytokine and chemokine profile from mouse UC-MSCs and human-derived MSCs

| Effect | Mouse UC-MSCs | Human MSCs |
| --- | --- | --- |
| VEGF | + | + [61, 62] |
| FGF2 | + | + [61] |
| Ang2 | + | + [62] |
| PDGF | + | + [62] |
| IL-6 | + | + [61, 62] |
| IL-10 | - | + [63][64] |
| iNOS | + | + [61] |
| SCF | + | + [61] |
| CXCL12 | + | + [61, 62] |
| TGFβ | + | + [61] |
| HGF | - | + [59, 61, 62] |
| GDNF | + | + [33] |
| BDNF | - | + [33] |
